# FIM, a Novel FTIR-Based Imaging Method for High Throughput Locomotion Analysis

**DOI:** 10.1371/journal.pone.0053963

**Published:** 2013-01-21

**Authors:** Benjamin Risse, Silke Thomas, Nils Otto, Tim Löpmeier, Dimitar Valkov, Xiaoyi Jiang, Christian Klämbt

**Affiliations:** 1 Institute for Neurobiology, University of Münster, Münster, Germany; 2 Institute for Computer Science, University of Münster, Münster, Germany; Imperial College London, United Kingdom

## Abstract

We designed a novel imaging technique based on frustrated total internal reflection (FTIR) to obtain high resolution and high contrast movies. This FTIR-based Imaging Method (FIM) is suitable for a wide range of biological applications and a wide range of organisms. It operates at all wavelengths permitting the in vivo detection of fluorescent proteins. To demonstrate the benefits of FIM, we analyzed large groups of crawling Drosophila larvae. The number of analyzable locomotion tracks was increased by implementing a new software module capable of preserving larval identity during most collision events. This module is integrated in our new tracking program named FIMTrack which subsequently extracts a number of features required for the analysis of complex locomotion phenotypes. FIM enables high throughput screening for even subtle behavioral phenotypes. We tested this newly developed setup by analyzing locomotion deficits caused by the glial knockdown of several genes. Suppression of *kinesin heavy chain* (*khc*) or *rab30* function led to contraction pattern or head sweeping defects, which escaped in previous analysis. Thus, FIM permits forward genetic screens aimed to unravel the neural basis of behavior.

## Introduction

Most animals have the ability to move and during evolution increasingly complex nervous systems allowed sophisticated locomotion control. Neural computing is put into operation by the interplay of a large number of neurons with numerous and specific interconnections and the non-neuronal cells of the nervous system, the glial cells. To decipher the role of individual genes in controlling neural network function, model organisms such as Drosophila provide the advantage of easy cell specific manipulations [1]. However, a thorough understanding of behavior also requires the ability to quantitatively asses different locomotion patterns.

In Drosophila, locomotion of both, adults and larvae is guided by environmental cues and is pivotal for finding mating partners, pupariation spots or food [2]. However, tracking of freely flying Drosophila is a tantalizing task [3]–[6]. In contrast, larval crawling occurs in two dimensions at relatively low speed and crawling patterns of single larvae have been analyzed using elaborated microscope setups [7]–[9]. In principle, larval movement can also be documented by a simpler camera setup. However, recording of crawling larvae requires high contrast images, which can be obtained following sophisticated illumination protocols or dye applications [10]–[13]. For conventional, relatively low resolution tracking of larval locomotion, larvae are illuminated by incident or transmitted light and monitored by cameras with appropriate filters. This is technically challenging due to the semi-translucent body of these small animals. In addition, the observation of larvae is complicated by light reflections caused by the tracking surface. Thus, illumination problems aggravate faithful recordings of larval crawling paths and the poor signal to noise ratio hinders subsequent computer-based analysis.

In addition, the existing tracking programs generally loose trajectories of colliding or pausing larvae (e.g. EthoVision tracker [14], Multi-Worm-Tracker [15], MAGAT [10], Image Pro Analyzer [MediaCybernetics], Actual Track [Actual Analytics]). The often used Multi-Worm-Tracker works online. Several custom-made tracking software modules have been adjusted for specific experimental questions, usually focussing on single larval movements [16] requiring extensive user input [8]. A program has been reported to solve collision events, however, in this case only the midpoint of the animal is taken into account [17].

To improve tracking of Drosophila larvae and to implement the simultaneous analysis of multiple animals we have developed a novel 2D-monitoring system based on Frustrated Total Internal Reflection using infrared light (FTIR). This new imaging approach named FIM (FTIR-based Imaging Method), provides an unprecedented high contrast view on crawling animals and even allows to image internal organs. Experimental and control Drosophila larvae can be recorded at the same time and the respective genotypes can be distinguished by GFP expression. Furthermore, FIM-imaging is the basis for computer based head recognition and enables the preservation of larval identity during collision events, which is implemented in a new tracking program FIMTrack. In summary, FIM together with the optimized tracking software facilitates analysis of larval locomotion and will simplify genetic screening procedures.

## Results

### FIM-imaging of larvae

**Figure 1:**
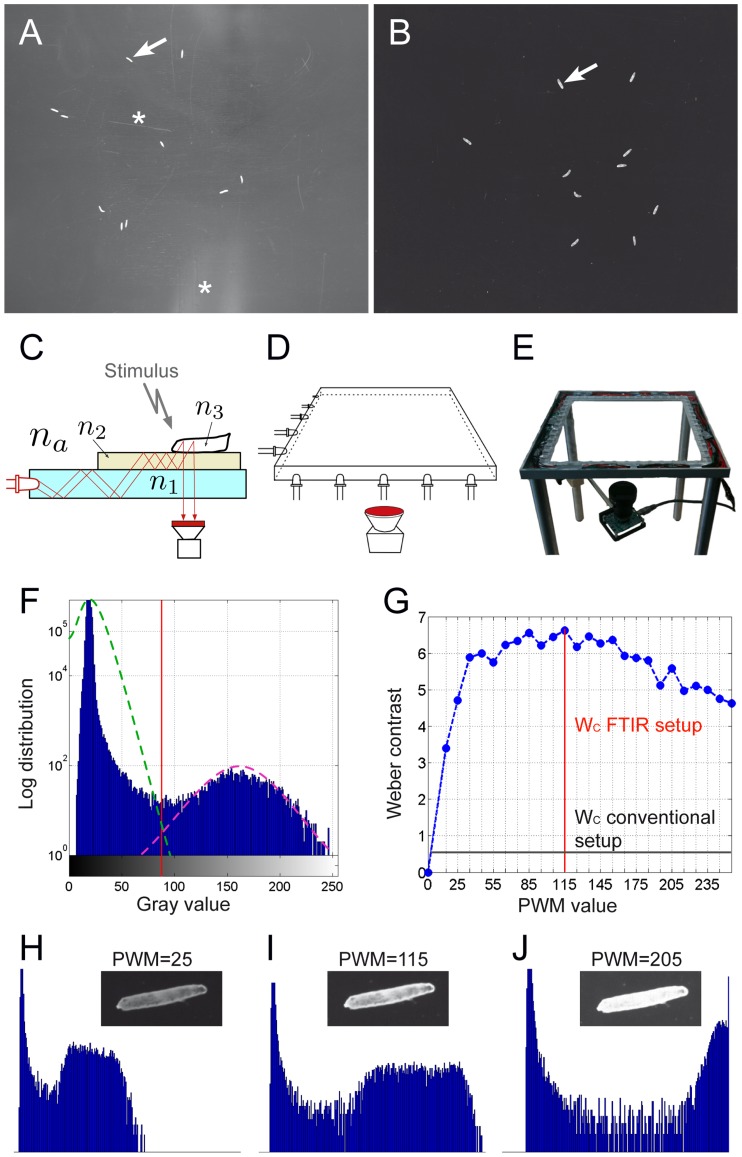
The FIM setup. (**A**) Image of 10 larvae (arrow) imaged in a conventional setup. The asterisks denote scratches and reflections in the tracking surface. (**B**) Image of 10 larvae (arrow) imaged in the FIM setup. Note the high contrast. (**C**) The principle of frustrated total internal reflection. n1 to n3 indicate different refractory indices, an acrylic glass plate is flooded with infrared light (indicated by red lines). The camera is mounted below the tracking table. (**D**) Schematic drawing of the setup. (**E**) Image of the tracking table. (**F**) Histogram of the image shown in (b). (**G**) Comparison of Weber contrast (W_c_) obtained in the conventional and the FIM setup. The pulse-width modulation (PWM) is plotted against the Weber contrast. (**H–J**) Histogram taken at different PWM values as indicated.

To analyze moving Drosophila larvae, both, incident and transmitted light can be used during recording and thus, the camera captures either light reflection or light absorption. The subsequent computer aided tracking of motion paths includes the extraction of circumferences and locomotion features over time. The quality of the tracking is strongly dependent on the quality of the acquired images, and therefore, lighting conditions need to be carefully adjusted to obtain high contrast images [11]. In a conventional illumination and recording setup, we used a 4 MP camera to image Drosophila third instar larvae in a resolution of about 40 pixel per larval length in a 25 cm×25 cm test arena (Fig. 1A). Frequently, the relatively low contrast in these movies as well as random light reflections at the crawling surface generate problems in automatic image tracking.

We therefore developed a new image acquisition technique to obtain high contrast images with no constrains by external lighting conditions (Fig. 1B). Instead of directly illuminating crawling larvae, we use frustrated total internal reflection (FTIR) to determine the contact surface between the animal and the substrate (Fig. 1C–E). In the FIM setup, an acrylic glass plate is flooded with infrared light. Due to the differences in the refractive indices of acrylic glass and air, it is completely reflected at the glass/air boundary (Fig. 1C). To provide a moist crawling environment we add a thin agar layer. According to Snell's law, the light enters the agar layer since its refractive index (n_2_) is higher than the refractive index of the acrylic glass (n_1_, Fig. 1C). The larvae have an even higher optical density resulting in a higher refractive index (n_3_), and thus, reflection is frustrated at the agar/larva interface and light enters the larval body. Here, light is reflected and since the reflection angle is smaller than the critical angle, the light passes through the different layers and can be detected by a camera equipped with an infrared filter (Fig. 1D, 1E). This setup is easy to assemble and does not require cost intensive equipment. Without any background subtraction it generates constant image quality superior to previous setups (Movie S1, S2, and S3).

### FIM provides high contrast data sets

In black and white images, contrast makes objects distinguishable from the background. Contrast is the ratio of luminance difference between foreground (μ_1_, i.e. larvae) and background (μ_0_) normalized by the average luminance. Assuming a bimodal gray value distribution, where one Gaussian describes foreground and the other Gaussian describes the background, Otsu's method can be used to determine the best possible threshold and, thus, μ_1_ and μ_0_ can be calculated as the mean values of these two Gaussians (Fig. 1F). Tracking multiple small animals on a wide surface leads to an unequal distribution of foreground and background pixels. Due to the large uniform background the average luminance is approximately equal to the background luminance (Weber contrast, *W_c_*).
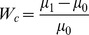
Whereas conventional imaging does not generate divisible grey value distributions, FIM provides a higher *W_c_* and results in a clear separation of fore- and background (Fig. 1F).

In the current FIM setup, only the intensity of the 4×24 infrared light emitting diodes (IR- LEDs), which are integrated into a 32 cm×32 cm acrylic glass plate need to be adjusted. To determine the optimal illumination intensity, the LEDs are regulated by a micro controller connected to the circuit (Materials and Methods). The power of the LEDs is controlled via pulse-width modulation (PWM) and even under full power no heating of the test arena is observed. Plotting *W_c_* against the PWM values results in a curve with a broad contrast maximum between 80 and 140 (Fig. 1G–J), demonstrating that FIM yields very high contrast images almost independent of illumination intensity. A similar calculation of the image contrast quality was obtained using Otsu's quality measure (data not shown). Since the camera is placed below the tracking table, additional stimuli can be easily incorporated into the setup (Fig. S1). In conclusion, FIM-imaging eliminates or reduces many of the previously encountered illumination problems on the physical level rather than using time consuming contrast enhancement techniques.

### FIM generates high resolution images

Since the IR light sources are integrated into the tracking plate, there is no need for further filter and light adjustments once the IR intensity is set. Importantly, the system performs a self-calibration to guarantee the best possible foreground/background segmentation based on the bimodal grey value distribution of the images (Materials and Methods). The overall brightness in the FIM setup is much smaller compared to incident or transmitted light situations. Therefore, the sensitivity of FIM allows an unprecedented detection of internal structures.

**Figure 2:**
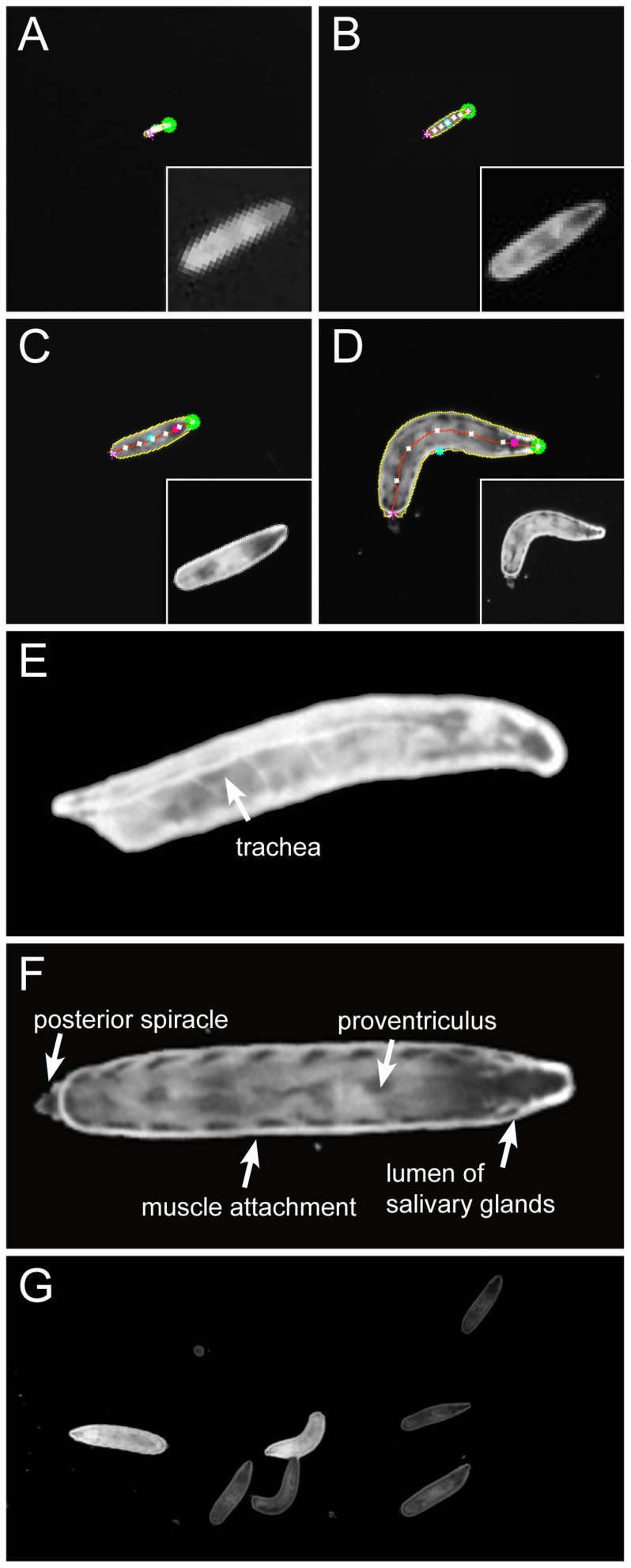
Recognition of inner structures. (**A–D**) Comparison of spatial resolution at different tracking arena sizes. The image shows the output of the tracker in true resolution. The green dot demarcates the moving tip of the animal. In the inlay the larvae are magnified to comparable sizes to show the spatial resolution. (**A**) Arena of 42.5 cm×42.5 cm; the size of a third instar larvae is 25 pixel per larval length. No automatic head recognition is possible. (**B**) Arena of 25 cm×25 cm; the size of a third instar larvae is 40 pixel. In about 60% of all frames the head can be identified. (**C**) Arena of 13 cm×13 cm; the size of a third instar larvae is 75 pixel. In 98% of all frames the head can be recognized. (**d**) Arena of 6 cm×6 cm; the size of a third instar larvae is 170 pixel. (**E,F**) Two 170 pixel images demonstrating the high resolution of FIM. Several inner structures are indicated. (**G**) Screen shot of a movie taken with illumination by 470 nm LEDs, seven larvae are shown, two of which express *daGal4* driven GFP.

To determine the best imaging conditions, we used a 4 MP camera with an infrared filter (Materials and Methods) and recorded crawling larvae at different spatial resolutions with a constant frame rate of 10 fps. Best results were obtained on 2 mm thick 0.8% agar. When resolution is set to represent each third instar larval length in 25 pixels, the image field is 42.5 cm×42.5 cm, however, no internal structures can be recognized (Fig. 2A). When resolution is set to 40 pixels per larval length, internal organs become visible. The head is defined as the largest dark spot at one end of the animal. It is identified in about 60% of the frames (verified manually by tracking 60 larvae over 550 frames, Fig. 2B). In the remaining frames the position of the head can be calculated based on the movement vector. The size of the tracking arena is now 25 cm×25 cm. When resolution is set to 75 pixels (arena size: 13 cm×13 cm), automated head recognition works in 98% of all frames (Fig. 2C). When resolution is set to 170 pixels per larval length (arena size: 6 cm×6 cm), many internal organs become visible and head recognition is further improved (Fig. 2D, 2E, 2F).

Moreover, the physical principles underlying FIM are working with different wavelengths and the use of UV-light easily allows the detection of GFP-expressing animals (Fig. 2G). In combination with specific chromosomes directing GFP-expression, FIM-imaging allows to identify a particular larva which makes it possible to simultaneously monitor control and mutant animals.

### Definition of tracking features

**Figure 3:**
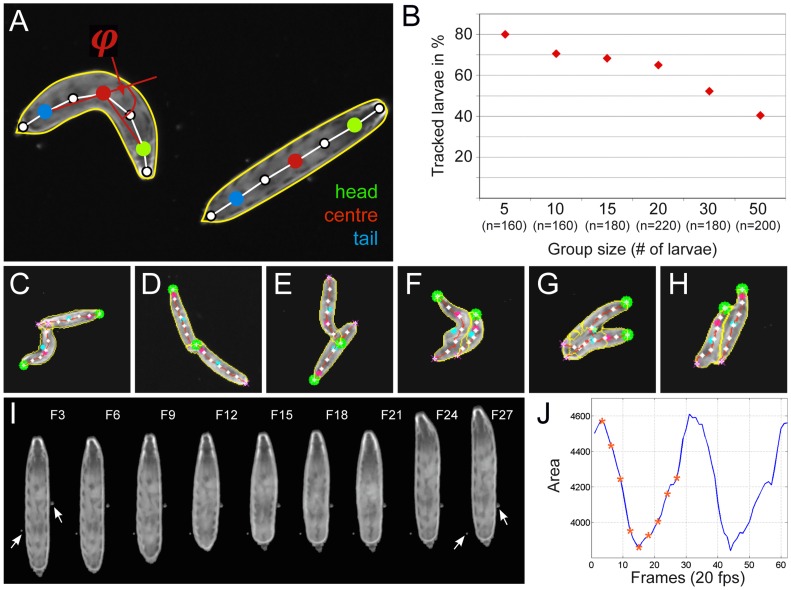
FIM-imaging allows extraction of several features. (**A**) Two larvae are shown in the 170 pixel per larval length resolution. The different features extracted are indicated. The spine is indicated by a white line. On the spine seven landmarks are positioned. The head (green dot), the center (red dot) and the tail (blue dot) are indicated. The contour line (yellow) and the bending angle φ are shown. (**B**) Plot of different group sizes against the percentage of completely tracked paths. The number of larvae tracked (n) is indicated. Recording for 3 minutes at 10 fps and 40 pixel larval length resolution. (**C–H**) Examples of larval collisions. (**C–E**) Moderately touching larvae can be separated. (**F–H**) In case of more intensive collision, the separation of larval contours is not perfect. (**I**) Stills of a movie showing a larval contraction wave. The numbers indicate the frames shown in (J). (**J**) The area covered by a larvae changes during contraction. Asterisks indicate the positions of the images shown in (I).

FIM allows high-quality recordings of larval movements. In a next step we established a program, FIMTrack, to extract and compute information from the movies. We delineate the circumference of the animal by a contour detection algorithm, calculate the spine and identify the head as the darkest blob at one end (Figs. 2, 3, Materials and Methods). Seven equidistant landmarks are defined on the spine (0 to 6). Landmark 1 serves as head position, landmark 3 is the center of the animal and landmark 5 defines the tail (Fig. 3A).

**Figure 4:**
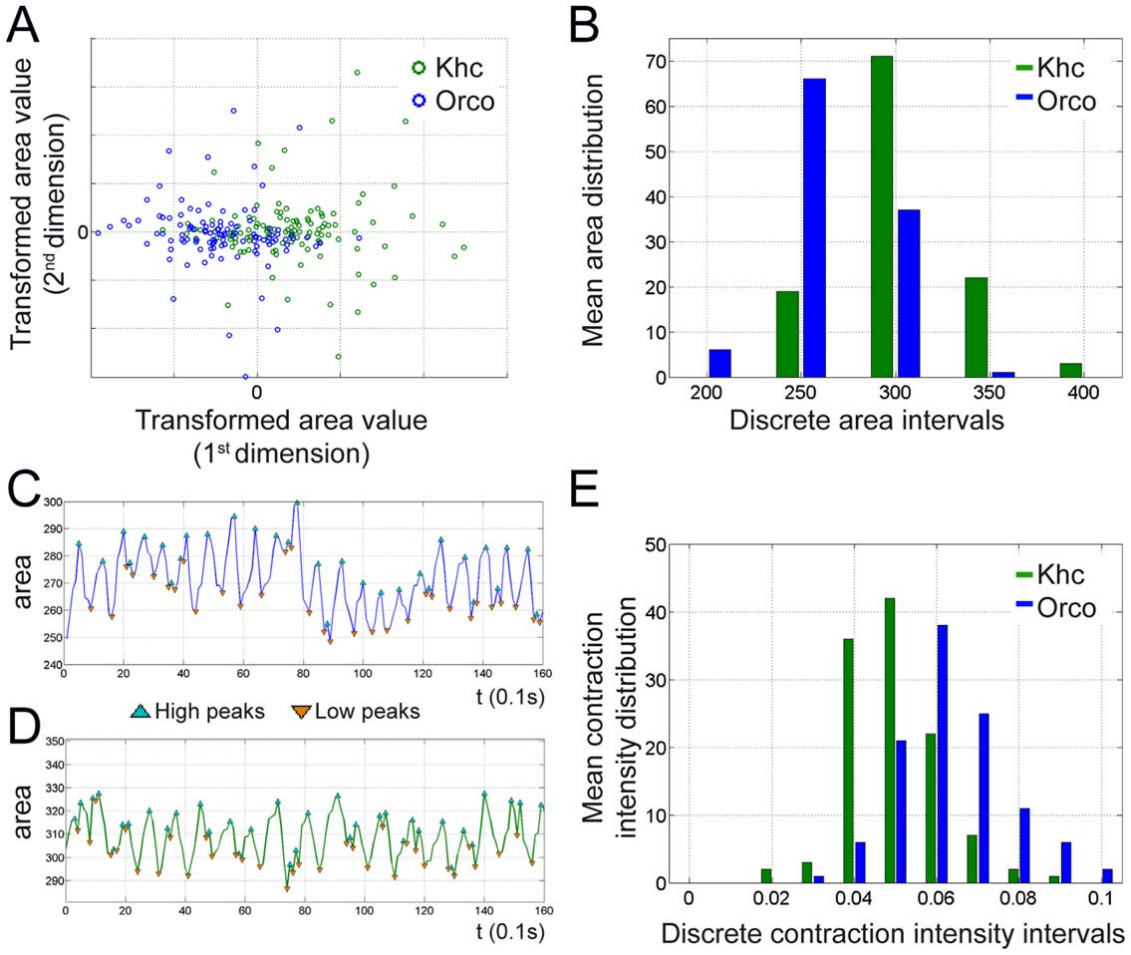
Glial suppression of *khc* reduces contractibility. (**A**) MDS analysis of area sizes (area^1800^ to area^2^) of individual larvae expressing *khc* dsRNA (green circles) or *Orco* dsRNA (blue circles) in all glial cells. (**B**) Discretized distribution of mean area sizes. The panglial knockdown of *khc* leads to bigger larvae. (**C,D**) Plot of area changes over time. (**C**) Panglial knockdown of *Orco*. (**D**) Panglial knockdown of *khc*. (**E**) Discretized contraction intensity intervals plotted against the mean contraction intensity. Upon *khc* knockdown reduced contraction intensity is observed.

The parameters allow to extract several features. The landmark 3 is used to calculate distance traveled and velocity. Landmarks 1,3,5 are used to extract the bending angle φ (Fig. 3A), which can be used to extract the bending rates of the larvae (verified by manually tracking 10 larvae over 2100 frames, see Materials and Methods). Larval head bending is also referred to as head sweeping [7], [8], head swinging [18] or head casting [19]. The contour defines the area occupied by the animal. A change of area is for example expected during peristaltic contraction movements [8], [20]. Indeed, this can be observed from a 40 pixel per larval length resolution onwards, highlighting the excellent resolution of FIM (Figs. 4C, 4D, 3I, 3J). It easily allows to detect the contraction wave which propagates from posterior to anterior through the larval body (Fig. 3I, 3J, Movie S2).

### Screening for larval locomotion phenotypes

In Drosophila, genetic tools allow the dissection of even complex phenotypic traits [1]. This, however, requires the analysis of many animals. An increase in the number of larvae crawling on the tracking arena is expected to result in a higher number of collision events. In general, the identity of the animals is lost during collisions and trajectories are fragmented.

To increase the number of analyzable paths, we aimed for a software module integrated in FIMTrack capable of keeping identities of colliding animals. In our current version, animals can be separated and identities are kept when larvae touch for less than 70% of their body length (Fig. 3C–H, Movie S4). However, during intense collisions the shape of the larvae can be distorted (Fig. 3F–H, Movie S5). When larvae crawl over each other or stay together for extended time periods, no separation can be calculated and the corresponding tracks are disrupted (Movie S6). By now, FIMTrack cannot resolve collisions involving more than two animals. In conclusion, these procedures allow to analyze a large number of tracks that cover the entire experimental time. If desired, fragmented tracks can be retrieved and also be used for calculation.

To determine the optimal population density of larvae, we set the imaging resolution to 40 pixel per larval length. Animals, reared in constant conditions, were placed in the middle of the arena and movement was monitored without any external stimulus for three minutes at 10 fps (see Materials and Methods). 80% of the animals that were tracked in groups of five could be followed from start to end (Fig. 3B). In contrast, only 41% of the animals that were tracked in groups of 50 could be followed from start to end due to more collisions (Fig. 3B). Intermediate values were determined for group sizes of 10, 15, 20 and 30 animals. In summary, the collision frequency shows an almost linear relation with the group size (Fig. 3B). For practical reasons we selected 15 larvae as the standard for screening purposes.

### Analysis of genes controlling larval locomotion

Animal locomotion is essential for finding optimal growth and survival conditions or finding mating partners [2]. It is guided by numerous external cues which are processed and integrated in still unknown neural networks. To decipher the molecular bases of complex behavioral tasks, a computational analysis of locomotion paths is essential. To demonstrate the feasibility of FIM in this respect, we selected 10 genes for further analysis. These genes were previously identified in a screen for genes required in glial cells to allow normal adult locomotion [21]. In addition, we analyzed the gene *Odorant receptor co-receptor (Orco)* as a negative control, since to our knowledge, *Orco* is not required in glial cells and *Orco* knockdown animals behave like *w^1118^* animals (data not shown). The function of all genes was suppressed by RNA interference using the panglial Gal4 driver *repoGal4*. From 100 to 120 complete larval trajectories (three minutes each), the following parameters were determined by FIMTrack: area of the animal, bending angle and total distance traveled (Table 1).

**Table 1:** Summary of a small RNAi based screen.

silenced gene	area (pxl)	bending angle (degree)	total distance (mm)
***Orco*** (n = 110)	261.75±11.89	0.12±5.22	296.84±28.87
***khc*** (n = 115)	**305.5**±13.44	0.21±4.97	**235.37**±31.07
***rab30*** (n = 112)	280.25±17.9	0.21±**8.72**	**230.26**±34.18
***rab21*** (n = 121)	269±8.25	0.27±5.0	273.35±34.16
***rab9*** (n = 117)	267.5±9.51	0.25±4.4	290.9±28.82
***fas2*** (n = 113)	264±15.11	0.06±4.2	270.18±28.81
***fray*** (n = 98)	255.75±10.26	0.2±4.35	277.18±31.07
***mGluRA*** (n = 119)	266.5±11.8	0.19±4.03	279.9±34.43
***ppk12*** (n = 113)	266.5±10.96	0.0±3.99	277.84±36.43
***cac*** (n = 104)	266.5±13.77	0.23±4.51	295.75±29.87

The activity of 10 genes was silenced specifically in all Drosophila glial cells using the *repoGal4* driver element. 10 times 15 animals were tracked for 3 minutes at 10 fps. The total number of complete tracks is indicated. We determined the median values for area size in pixel, bending angle φ in degree and the total crawling distance in mm. The standard deviation is indicated in each case. Values that differ significantly from the control (*Orco*) are printed in bold.

Surprisingly, glial knockdown of *kinesin heavy chain* (*khc*) led to a larger average surface size (Table 1). Indeed, multi dimensional scaling (MDS) analysis of the area distribution demonstrated a significant difference (Fig. 4A, Materials and Methods). This indicates that either the *khc* knockdown larvae are larger and/or their contraction ability is impaired. We then plotted all individual area sizes of *khc* and *Orco* knockdown animals and noted that *khc* knockdown resulted in larger larvae (Fig. 4B). To address, whether this size difference is caused by less contraction in *khc* knockdown animals or native larval length we generated maximally relaxed larvae by submersing them in ethanol for 30 minutes. Subsequently, we determined the length, surface area and the weight. In all parameters *khc* knockdown larvae were about 12% larger (data not shown).

The oscillation frequency of the area size revealed a 10% decrease upon panglial knockdown of *khc* (Fig. 4C, 4D; median frequency of 110 or 115 animals imaged over 3 minutes: *repo≫Orco^dsRNA^* 0.64 seconds per contraction, *repo≫khc^dsRNA^* 0.72 seconds per contraction). In addition, the contraction intensity is altered. The contraction intensity is reflected by the mean difference between the maximal surface area and the minimal surface area normalized by the median surface area of 140 dead larvae for each genotype. Plotting the discretized contraction intensities clearly indicates that *khc* silencing results in less intensive body contractions (Fig. 4E). This contraction phenotype may explain why *khc* knockdown larvae also travel 20% less in total distance (Table 1).

**Figure 5:**
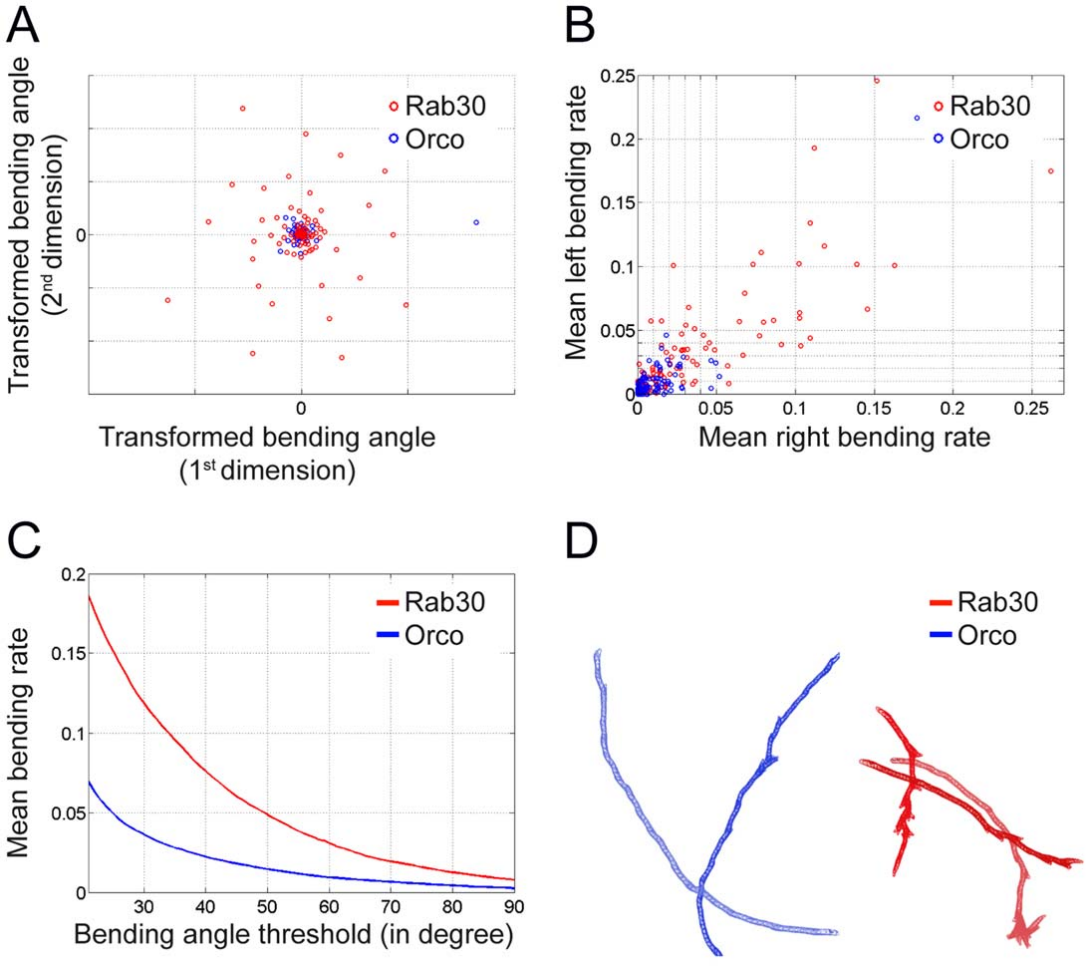
Glial suppression of *rab30* increases head bending intensity and frequency. (**A**) MDS analysis of bending angles (angle^1800^ to angle^2^) of individual larvae expressing *rab30* dsRNA (red circles) or *Orco* dsRNA (blue circles) in all glial cells. (**B**) Plot of mean bending rate (left vs. right) for every animal. A turning tendency to the right can be seen. (**C**) Plot of bending angle threshold against mean bending rate per genotype. (**D**) Trajectories of larvae upon glial suppression of *Orco* (blue) and *rab30* (red).

A similar reduction in crawling speed was noted upon *rab30* knockdown, which is mis-distributed in glial cells upon *khc* suppression (Table 1, [21]). The area size of *rab30* depleted animals appears only slightly increased. Thus, the reduction in crawling speed must be explained by additional means. An increased standard deviation in the bending angle φ and MDS analysis shows a clear separation of *rab30* knockdown and control animals (Fig. 5A). To further study bending intensity we plotted the bending rate (φ larger then 40° [8]) towards the left and the right side (Fig. 5B). This shows that *rab30* knockdown animals bend more often with a slight preference towards the right side. Interestingly, we noted that almost all genotypes show a preferred bending towards the right side (Table 1). In addition, we plotted the normalized overall number of bendings against increasing angle thresholds from 20° to 90° demonstrating a particular increase in the number of small head bends (Fig. 5C, 5D), which might explain the reduced migratory distance.

In conclusion, FIM allows to easily detect even subtle phenotypic variants. Thus, it is well suited for high throughput approaches required for forward genetics.

## Discussion

To decipher complex animal behavior, large numbers of individuals must be observed at high resolution and automated procedures have to be available for statistical analysis. Previously, insufficient image quality made computer aided image analysis difficult. To solve this challenge and to obtain images at high contrast and resolution, we have introduced FIM to monitor Drosophila third instar larvae. In addition, we established FIMTrack to extract many features used to mathematically dissect complex animal behavior.

FIM is a simple, robust and reliable methodology, which provides an image quality comparable to an automated microscope setup [7], [8]. Without further adjustments it can be used to monitor all larval stages, foot prints of adult Drosophila, *C. elegans*, planarian flatworms or the growth of *Arabidopsis thaliana* seedlings (Fig. S2). Moreover, it allows the simultaneous analysis of a large number of animals in a wide tracking arena. Here we demonstrate that for screening purposes, an arena size of 25 cm×25 cm is suitable with a 4 MP camera. Since one side of the FIM setup is completely accessible and the tracking arena is translucent, additional equipment and external stimuli such as a temperature gradient, olfactory stimuli or light patterns can be easily applied (Fig. S1). Moreover, the physical principles underlying FIM are working with different wavelengths. The use of UV-light allows the detection of GFP-expressing animals. Since the tracking software recognizes a particular larval identity, only rare UV-pulses are sufficient to identify GFP-expressing animals. Using GFP-tagged chromosomes, experimental larvae and their control siblings can be monitored at the same time (Fig. 2G). In addition, the expression of GFP (or infrared fluorescent proteins [22]) in defined tissues could aid the analysis of more complex behavioral traits in the future.

The wealth in high contrast images allows to extract several features from the moving animals. The identification of the head helps to determine the orientation of the animals.

To obtain the largest number of trackable trajectories in minimal time, we tested different group sizes of larvae. The number of larval collisions increases almost linear with the number of tracked animals. This suggests that no community effects alter the collision frequency and thus, measurements in larger animal groups can be conducted (data not shown).

Here we have exemplarily extracted larval size, bending angle and traveled distance and performed several statistical analyses to identify significant data sets. Further features such as stop and go phases, types of head casts or body curvature can be calculated from the existing values and will aid further dissection of animal behavior. A demonstration of the visualization of stop and go phases and the bending directionality is shown in Movie S7.

Using a conventional tracking setup, we have previously shown that glial knockdown of *khc* renders the animals slower but no further characterization of the phenotype was possible [21]. Applying electrophysiology, we had demonstrated a reduced conductance velocity in peripheral nerves, but had not seen any change in the contraction patterns [21]. Using FIM at a relatively low resolution of only 40 pixels per larvae we could now extract a number of features explaining the reduced crawling distance by alterations in the contraction pattern. *khc* encodes a motor protein that carries vesicular compartments along microtubules. Some of these vesicles are marked by Rab30 [21] and indeed FIM detects alterations in larval locomotion upon glial specific knockdown of *rab30*. Interestingly, in these animals the reduction of crawling speed is achieved by an increase of head bending intensity and a slight change in contraction intensity. This demonstrates that *rab30* also functions independently of *khc*. Thus, FIM is well suited to screen complex behavioral traits.

In this study the average duration for a contraction cycle is about 0.7 seconds compared to duration lengths between 1 and 2.4 seconds found by others [20], [23]–[25]. Interestingly, we determined an average contraction duration of 1.4 seconds per contraction in a different wild type background [26] (data not shown), highlighting the need for carefully chosen controls and solid statistical analyses. We expect that the analysis of complex animal movement patterns will greatly benefit from the straightforward design of the FIM setup and the further implementation of software modules such as a feature selection tool. Thus, FIM facilitates high throughput analyses of complex behavioral traits.

## Materials and Methods

### Drosophila genetics

The experimental animals were kept at 25°C at 65% humidity. For all knockdown experiments 45 virgins were crossed to 7–10 males of the desired genotype and transferred to fresh food every two days. Third instar larvae were always selected five days after egg laying in the wandering third instar stage.

### Hardware

The tracking stage is a table with an acrylic glass table plate (Figure 1). IR LEDs (Hewlet Packard HSDL 4230) are plunged into the edges of the plate and connected to a custom made circuit. Even under full power, the LEDs do not warm up the tracking arena (after 2 hours of full power no temperature change was detected on the entire tracking surface using a TROTEC BP20 laser thermometer and a VOLTCRAFT VC150 digital multimeter). Depending on the size and the associated weight of the model organism, the used intensity of IR light can be limited on a hardware level by using a higher or lower amperage. To precisely adjust the intensity of the LEDs an Arduino Mega 2560 micro controller (MC) is connected to the circuit. The adjustment can be both, turning several LEDs on and off and controlling the power of the LEDs by setting the pulse-width modulation (PWM) to a value between {0,255}, where 0 indicates no power and 255 indicates full power. A tool written in C++ controls illumination. Once the illumination intensity is set, there is no need for further light intensity adjustments and the controller will supply the circuit with the correct settings even if it is not plugged to the computer (i.e. the illumination setting program is stored in the program memory of the MC). Especially no diffusors or other filters are used to produce uniform and homogeneous lightning conditions. Further details of the setup are available upon request.

We evaluated our setup with three different cameras (a Point Grey Dragonfly 2 (DR2-13S2M/C-CS) camera with a Tamron 3.0–8 mm (13VM308AS) lens; a QImaging 1394 firewire (01- QIC-F-M-12 MONO 12 Bit) camera with a depth of 8 bit and a resolution of 1392×1040 pixels and a Tamron T-23FM16SP 16 mm lens and a Basler ace (acA2040-180 km) with a Kowa 16 mm (LM16HC-SW lens) to demonstrate that FIM is independent from the general image acquisition. All cameras were equipped with an appropriate IR filter (Schneider IR Pass Filter IF 093 with 825 nm cut-on wave-length). All images used in this study were acquired using the Basler camera.

The firewire cameras and the micro controller are connected to a conventional computer (Apple iMac 27″, 4 GB RAM, 2.7 GHz Quad-Core Intel i5, AMD Radeon HD 6770M 512 MB GDDR5). To gather high resolution images from the Basler camera we used a Fujitsu Celsius W510 Power Workstation with an Intel Xeon E-1275 3.4 GHz CPU, 16 GB DDR3-1333 RAM, a NVIDIA Quadro 4000 2 GB graphics card and a Matrox Solios eV (MDR) SOL 2M EV CLF L framegrabber.

### Image quality evaluation techniques and parameters

To measure the contrast we used grey-level histogram based variance analysis. Especially Otsu's thresholding will be examined since its terminology and parameters are most established in Computer Vision. Assuming bimodal gray-level distribution, the foreground (i.e. the objects of interest) and background can be assumed as two distinguishable Gaussians with its respective maxima representing the mean foreground and background intensity. Each pixel belongs to either the background C_0_ or the foreground C_1_. The central statistics to evaluate the quality of the contrast is the background and object variance given by:
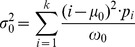
and
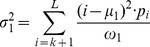
using L discrete gray values and class mean values
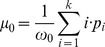
and
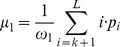
class occurrences
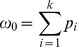
and
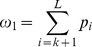
and normalized histograms p_i_. The value k∈{1, 2, …, L} is the threshold indicating that all pixel values x≤k belong to the background and all values x≥k+1 belong to the objects of interest (or vice versa). Then the *within-class variance* is defined as
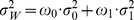
and the between-class variance is defined as
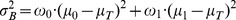
with
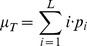
Following Otsu a measure of separability is given by
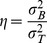
using
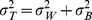
Maximizing η is the central criterion for automatic threshold computation and is equivalent to minimizing the within-class variance. Since many image quality measures for automatic target recognition relay on an explicit distinction between targets (i.e. foreground) and background, we will use Otsu's threshold estimation to distinguish between C_0_ and C_1_ in the following analysis methods.

In black and white images contrast is the difference in luminance, which makes objects distinguishable. The frequent definitions of contrast are based on ratios like:
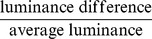
Tracking multiple small animals on a wide surface leads to an unequal distribution of foreground and background pixels. Assuming a large uniform background the average luminance is approximately equal to the background luminance. Since Otsu's method leads to the optimal threshold in a bimodal distribution, this threshold can be used to separate the histograms into foreground and background distributions. *Weber contrast* measures the quality of the contrast given an unequal distribution, where most of the pixels belong to the background:
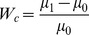
μ_1_ and μ_0_ represent the luminance of the foreground and the background respectively.

### Software

All statistical graphics and calculations are created using a Matlab programming environment.

#### LED Controller

The program to control the intensity of the MC is written in C++. The optimal PWM value calculation uses Otsu's quality measure η to quantify best possible illumination [27]. For every PWM value p between 1 and 255 (with p = 1 indicates a poor illumination and p = 255 a bright illumination) we acquire an image and calculate both, the optimal gray value threshold k and the quality measure η:
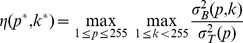
where 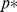, 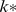 lead to the overall maximum. Therefore 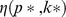 indicates best possible image quality to separate the bimodal foreground background gray value distribution and 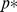 is the best possible PWM value (i.e. illumination).

#### FIMTrack Software

We implemented a custom tracking program, called FIMTrack, employing the OpenCV (v2.4) library for image processing and Qt (v4.8) library for the graphical user interface. FIM provides a constant image quality (i.e. contrast) so that batch processing is easily possible. The flow of the tracking software is as follows: The current frame is segmented using simple thresholding, all remaining pixels belong to the foreground. Then, the contour is calculated using an established algorithm [28]. An additional size threshold operation rejects contours without sufficient sizes. All remaining objects are assumed to be larval objects and are tracked over time. To assign larvae between consecutive time steps we compare both, the euclidean distance between the centers of mass and the size of the overlapping contours.

For every larva we calculate the following parameters in each frame: the spine represented by a spline, several (x,y)-landmarks along the spline (usually seven, including head, middle and tail landmark), angles between the landmarks and surface area of the contour. These parameters are used to calculate the following time variant features for the head middle and tail landmark: the distance, accumulated distance and distance to origin, the velocity (analogous to the distance with meaningful units) and acceleration. We identify the anterior ending (i.e. the head) by detecting the biggest dark region inside the contour.

The spline is calculated by deploying several random points inside the eroded contour. Then we calculate the shortest line between the random and the nearest contour points. The centers of these lines are sorted and passed to a least square spline fitting routine [29].

#### Separation of colliding larval tracks

To increase the number of analyzable paths, we have developed a collision routine which is able to resolve slight collisions rather than discarding all colliding animals or dividing tracks. If the distance between two individuals gets to small so that the two contours merge to one bigger blob, a collision handling routine is applied to these larvae: Assuming the collision occurs in frame t, then the movement vectors v_1_ and v_2_ between (t-2) and (t-1) of the colliding larvae are used to estimate the hypothetical positions at time step t by moving the contours from time step (t-1) in the direction of v_1_ (for the first larva) and v_2_ (for the second larva). All uniquely assignable pixels covered by these hypothetic contour positions are assigned to the respective larva. All remaining pixels are assigned via nearest neighbor to the closest contour in frame t. Since we capture the images with a relatively high frame rate (10 fps) compared to the slow movement speed, slight and short collisions can be resolved. The longer a collision continues, the more of the larval body lengths merge and the less accurate the collision resolution gets. Therefore, a collision flag in the output table informs the user about all collisions during the tracking procedure for each larva and time step, so that these time steps can be neglected in later analysis rather than discarding the animals or initialize new larvae and thereby divide the tracks.

#### Validation of FIMTrack results

FIMTrack has two modes: the actual tracking mode and an additional “view results” mode. Here, the output files can be reloaded and projected on the movie in three independent views: The overall view, the detail view and the table view. The overall view is an overlay of the tracking result (i.e. the trajectories) and the movie. In the detail view, the user is able to select a subset of the larvae and features which is then drawn directly on the images using full image resolution. The table view displays all acquired parameters for all selected larvae in a table. Since all views are accessible by registers, the user can easily switch between the views.

We validated all calculated features by manually inspecting movies using the “view results” mode. In addition, we tracked synthetic data and validated the results in the same manner. In a next step, we compared tracking results of FIMTrack with the Image Pro Analyzer. FIMTrack calculated the same momentum trajectories as the commercially available software tool. To confirm all features extracted by FIMTrack and its consistency, we developed an additional validation Matlab tool. This tool plots an arbitrary subset of features and larvae to find irregularities or cross-compare influences of several parameters between each other (e.g. by plotting distance to nearest neighbor and collision flag simultaneously).

In addition, more complex features like bending angle and stop and go phases were validated manually. Results generated for bending angle were confirmed by comparing manually generated results with the results of FIMTrack (movie of 10 larvae for >2000 frames). The bending angle was categorized in four discrete classes: no bending (x<∼20°), slight bending (∼20°<x<∼30°), average bending (∼30°<x<∼50°) and strong bending (x>∼50°). This ground truth data contained ∼460 slight, ∼470 average and ∼630 strong bending events. All discrete average and strong bending events could be detected by FIMTrack. Due to the chosen thresholding parameters not all slight bending events are identified correctly. Stop and go segmentation (see below) was validated using a custom Matlab script, plotting ‘stop’, ‘stop:left’, ‘stop:right’ and ‘go’ close to the respective larvae. The ‘left’/‘right’ bending is only indicated for average or strong bending events. The resulting movies were validated manually. Depending on the thresholds, the false positive rate for stop and go varies. No false positive go phases were detected upon validation applying the below mentioned parameters. All mentioned validation steps led to satisfiable results.

#### Stop and Go Segmentation

To separate the tracks into stop and go phases, we implemented a Matlab tool adding this features as binary indicator matrix to the overall feature sets. A go is defined as a phase of movement over t_go_ time steps with a movement speed above a velocity v (of the momentum of the contour) and with a body bending angle below β (t_go_, v and β can be set by the user). We use 10 fps temporal resolution to acquire smooth features (e.g. area values to calculate the peristaltic). The noise and the crawled distance between two adjacent frames is difficult to discriminate at this resolution(1 to 3 pixel given an 40 pixel larval length resolution for both, noise and traveled distance). We reduce the temporal resolution to distinguish stop and go phases. Therefore, we recalculate the movement speed for an increased time span t_window_ and apply a suitable speed threshold. For our experiments, a speed window t_window_ = 15 (1.5 fps temporal resolution), v = 10 (pixel per 1.5 frames) and bending angle threshold β = 20° lead to good results (see Movie S7). We smoothed the go phases by setting all t_go_ = 7, indicating that at least 7 frames must belong to an uninterrupted phase. The stop phase matrix is the logical not of the go phase matrix.

#### MDS

The tracking result is a table containing all tracked features. The rows represent time steps and columns represent animals. N targets tracked over T time steps result in (TxN) dimensional tables for each feature. Thus, every feature for every larva is stored in a N-dimensional vector and all animals can be represented in an N-dimensional feature space. Subsequently, N-dimensional feature space is projected into two dimensions using multidimensional scaling (MDS) deploying the Matlab programming environment [21], [30].

## Supporting Information

Figure S1 **Integration of external stimuli.** (**A**) Integration of a light pattern can be easily achieved by a conventional LCD projector placed below the FIM setup. (**B**) When second instar larvae are enclosed by a light ring, movement is confined to the dark spot. (**C**) To generate a temperature gradient we placed a metal plate with a temperature gradient of 0.8°C/cm 2 mm above the tracking arena. The tracking arena is equilibrated and the temperature on the agar is controlled. (**D**) Typical tracking pattern of wild type larvae in a temperature gradient (left 18.5°C to 33°C).
(TIFF)

Figure S2 **FIM offers a wide range of applications.** Examples of recording of different species. (**A**) Planarian flatworm. (**B**) Third instar Drosophila larva. (**C**) Second instar Drosophila larva. (**D**) First instar Drosophila larva. (**E**) Adult *C. elegans*. (**F**) Adult Drosophila. Note the bright footprints. All recordings were done at the same spatial resolution (third instar larva size 170 pixel) with 10 fps except for (F), 30 fps. Every fifth frame is shown. (**G**) Arabidopsis seedling.
(TIFF)

Movie S1 **FIM-imaging of multiple Drosophila larvae.** Group of crawling third instar larvae at 40 pixel per larval length with 10 fps recorded for 7.5 seconds.
(AVI)

Movie S2 **High resolution FIM.** Crawling larva at 170 pixel per larval length with 20 fps recorded for 2.25 seconds but shown at 5 fps.
(AVI)

Movie S3 **Integration of light stimuli in FIM-imaging.** Group of crawling third instar larvae at 40 pixel per larval length with 10 fps recorded for 28 seconds shown at 40 fps. Room illumination (350 lx) is turned on between frame 66 and frame 207.
(AVI)

Movie S4 **Larvae engaged in slight collisions.** Two colliding third instar larvae at 40 pixel per larval length with 10 fps. Larval identity and contour are maintained. Movies are recorded from the FIMtrack reviewing tool. Larval contours are selectively superimposed just for the colliding animals.
(AVI)

Movie S5 **Larvae engaged in moderate collisions.** Two colliding third instar larvae at 40 pixel per larval length with 10 fps. Larval identity is maintained but contour is distorted. Movies are recorded from the FIMtrack reviewing tool. Larval contours are selectively superimposed just for the colliding animals.
(AVI)

Movie S6 **Larvae engaged in intensive collisions.** Two colliding third instar larvae at 40 pixel per larval length with 10 fps. Larval identity and contour are not maintained. Movies are recorded from the FIMtrack reviewing tool. Larval contours are selectively superimposed just for the colliding animals.
(AVI)

Movie S7 **Extraction of stop and go phases.** The movie shows a video with superimposed stop and go phases and bending directions used to validate the extraction these features. Parameters are set in a way, that no false positive go-phases are indicated. This, however, produces some false positive stop-phases.
(AVI)
