# Supplementary figures and images for: FIM, a Novel FTIR-Based Imaging Method for High Throughput Locomotion Analysis
Source: PLoS One. 2013 Jan 21;8(1):e53963. doi: 10.1371/journal.pone.0053963 (PMC3549958; doi:10.1371/journal.pone.0053963)

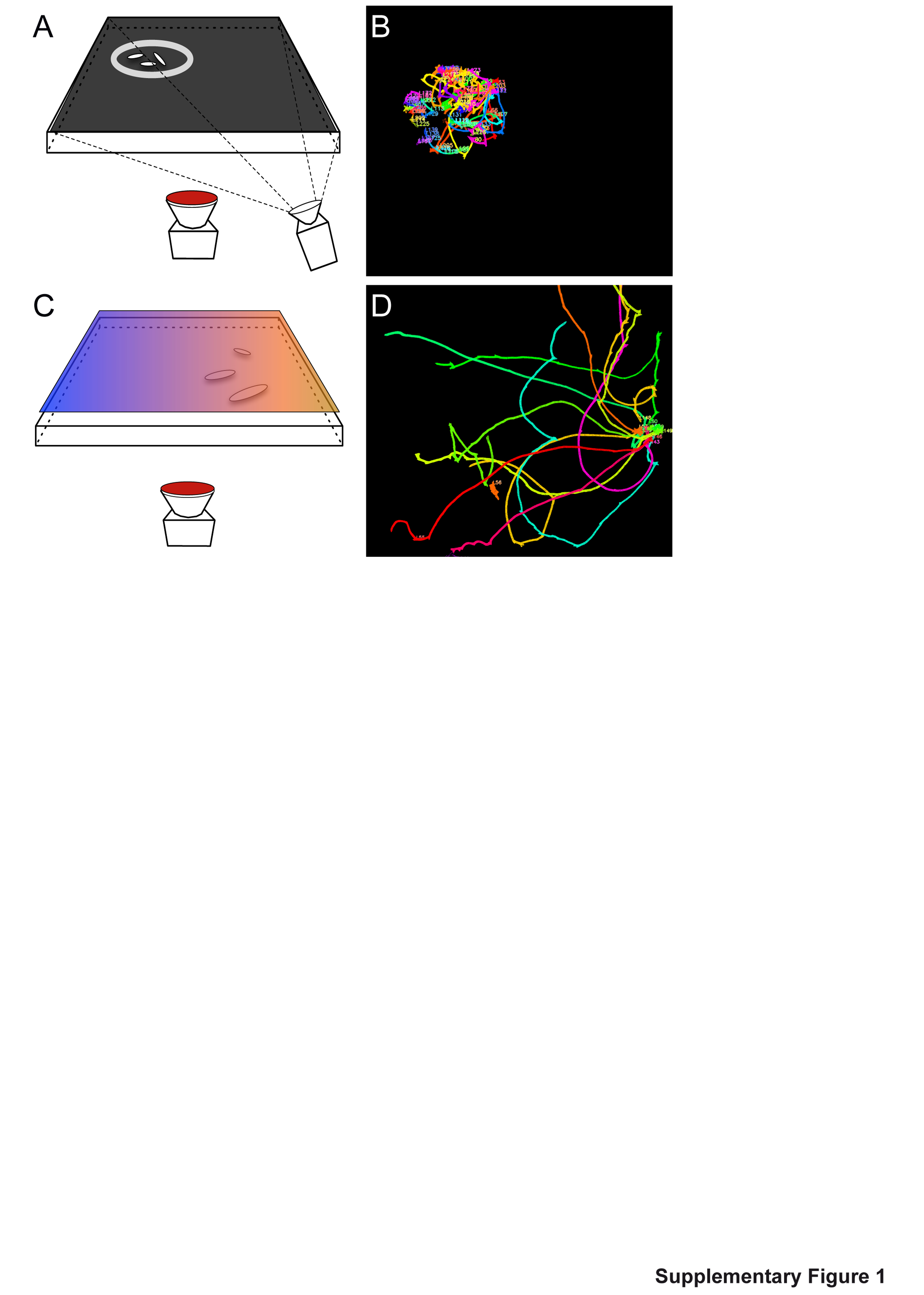

Supplement: Figure S1 — Integration of external stimuli. (A) Integration of a light pattern can be easily achieved by a conventional LCD projector placed below the FIM setup. (B) When second instar larvae are enclosed by a light ring, movement is confined to the dark spot. (C) To generate a temperature gradient we placed a metal plate with a temperature gradient of 0.8°C/cm 2 mm above the tracking arena. The tracking arena is equilibrated and the temperature on the agar is controlled. (D) Typical tracking pattern of wild type larvae in a temperature gradient (left 18.5°C to 33°C). (TIFF) [file pone.0053963.s001.tif]

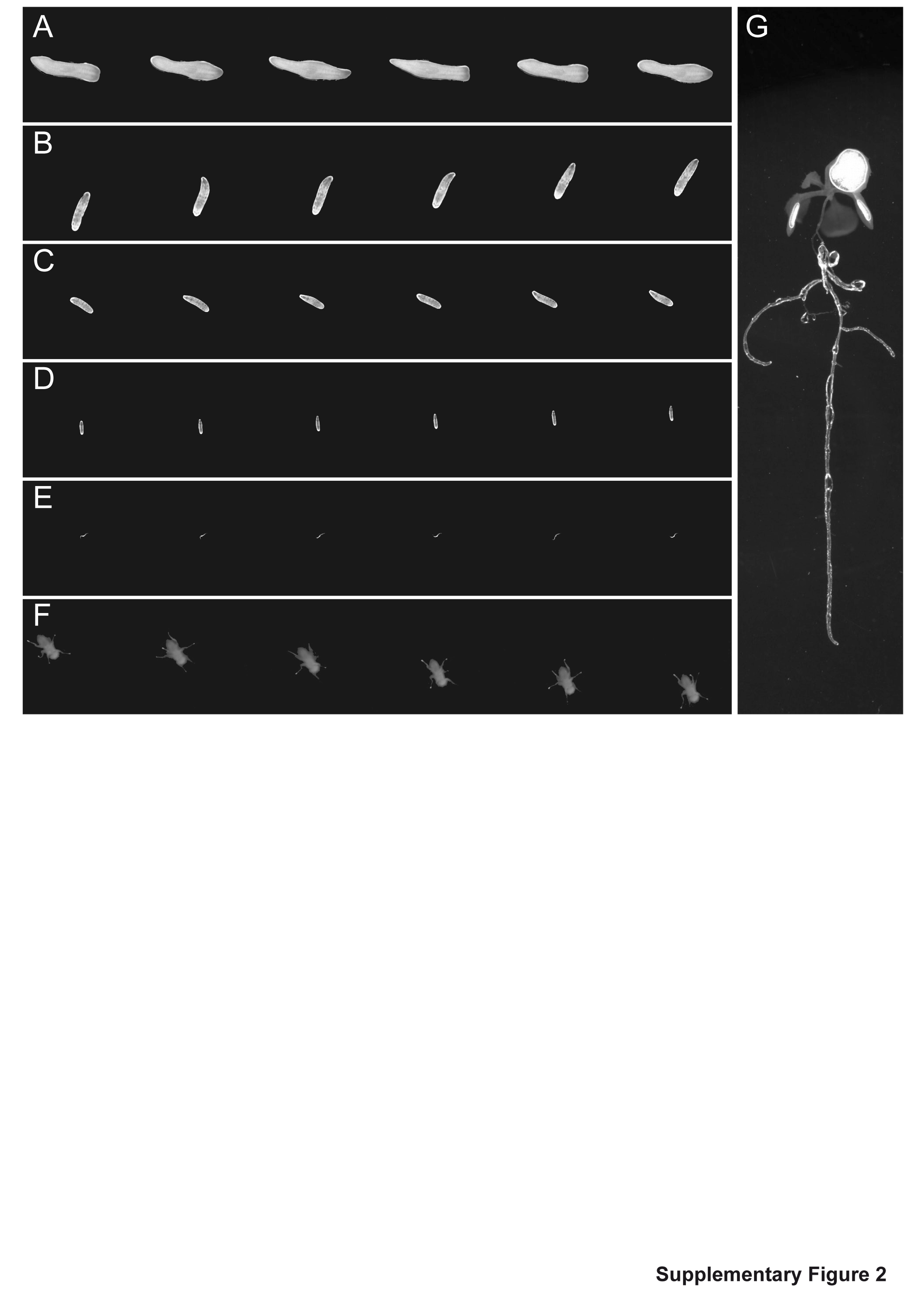

Supplement: Figure S2 — FIM offers a wide range of applications. Examples of recording of different species. (A) Planarian flatworm. (B) Third instar Drosophila larva. (C) Second instar Drosophila larva. (D) First instar Drosophila larva. (E) Adult C. elegans. (F) Adult Drosophila. Note the bright footprints. All recordings were done at the same spatial resolution (third instar larva size 170 pixel) with 10 fps except for (F), 30 fps. Every fifth frame is shown. (G) Arabidopsis seedling. (TIFF) [file pone.0053963.s002.tif]
